# Long noncoding RNA *EPCART* regulates translation through PI3K/AKT/mTOR pathway and PDCD4 in prostate cancer

**DOI:** 10.1038/s41417-024-00822-3

**Published:** 2024-08-15

**Authors:** Annika Kohvakka, Mina Sattari, Janika Nättinen, Ulla Aapola, Pavlína Gregorová, Teuvo L. J. Tammela, Hannu Uusitalo, L. Peter Sarin, Tapio Visakorpi, Leena Latonen

**Affiliations:** 1 Prostate Cancer Research Center, Faculty of Medicine and Health Technology, Tampere University and Tays Cancer Center, Tampere University Hospital, 33520 Tampere, Finland; 2 Eye and Vision Research Group, Faculty of Medicine and Health Technology, Tampere University, 33520 Tampere, Finland; 3 RNAcious Laboratory, Molecular and Integrative Biosciences Research Programme, Faculty of Biological and Environmental Sciences, University of Helsinki, 00014 Helsinki, Finland; 4 Department of Urology, Tampere University Hospital, Tampere, Finland; 5 Tays Eye Centre, Tampere University Hospital, 33520 Tampere, Finland; 6 HiLIFE Helsinki Institute of Life Science, University of Helsinki, 00014 Helsinki, Finland; 7 Fimlab Laboratories Ltd, Tampere University Hospital, 00014 Tampere, Finland; 8 Institute of Biomedicine, University of Eastern Finland, 70211 Kuopio, Finland

**Keywords:** Prostate cancer, Gene regulation, Tumour biomarkers

## Abstract

While hundreds of cancer-associated long noncoding RNAs (lncRNAs) have been discovered, their functional role in cancer cells is still largely a mystery. An increasing number of lncRNAs are recognized to function in the cytoplasm, e.g., as modulators of translation. Here, we investigated the detailed molecular identity and functional role of *EPCART*, a lncRNA we previously discovered to be a potential oncogene in prostate cancer (PCa). First, we interrogated the transcript structure of *EPCART* and then confirmed *EPCART* to be a non-peptide-coding lncRNA using in silico methods. Pathway analysis of differentially expressed protein-coding genes in *EPCART* knockout cells implied that *EPCART* modulates the translational machinery of PCa cells. *EPCART* was also largely located in the cytoplasm and at the sites of translation. With quantitative proteome analysis on *EPCART* knockout cells we discovered PDCD4, an inhibitor of protein translation, to be increased by *EPCART* reduction. Further studies indicated that the inhibitory effect of *EPCART* silencing on translation was mediated by reduced activation of AKT and inhibition of the mTORC1 pathway. Together, our findings identify *EPCART* as a translation-associated lncRNA that functions via modulation of the PI3K/AKT/mTORC1 pathway in PCa cells. Furthermore, we provide evidence for the prognostic potential of PDCD4 in PCa tumors in connection with *EPCART*.

## Introduction

Long noncoding RNAs (lncRNAs) are a heterogeneous group of regulatory RNAs that are >500 nt long RNAs with limited or no protein-coding abilities and are mostly generated by RNA-polymerase II [1]. A majority of lncRNAs are localized in the nucleus, although a large number of lncRNAs reside in the cytoplasm [2]. A large fraction of lncRNAs, especially cytoplasmic lncRNAs, have 5′ cap structures, 3′ poly-A tail, and multiple exons and are thus processed similarly to mRNAs, albeit with an altered efficiency [2].

While tens of thousands of lncRNAs have been detected, only a fraction of them have been fully characterized. Some lncRNAs have been found to have important roles in various biological functions, such as cancer formation and development [3, 4]. LncRNAs can act in the regulation of gene expression through several mechanisms [5]. Whereas nuclear lncRNAs function often as chromatin and transcriptional regulators, cytoplasmic lncRNAs may affect gene expression via modulation of mRNA stability, translation, and post-translational modifications [5].

In whole transcriptome studies of prostate cancer (PCa) tumor samples, hundreds of lncRNAs have been identified to be aberrantly expressed in primary and castration-resistant PCa (CRPC) [6–10]. Many of them have been shown to be regulated by PCa-associated transcription factors, most importantly by androgen receptor (AR) [11–13]. The functional role of these lncRNAs is poorly known, as only a few PCa-associated lncRNAs have been studied in depth [14]. Here, we studied the functional mechanism of *EPCART* (ERG-positive PC-associated androgen responsive transcript), which is a PCa-associated lncRNA that we have previously found to affect proliferation and migration of PCa cells in vitro and to associate with a more aggressive phenotype in PCa tumors [11]. We integrated whole transcriptomic and proteomics data of *EPCART* knockout cells to uncover the pathways and individual genes that could explain this oncogenic phenotype. Our results indicate that *EPCART* functions as a modulator of translation through AKT/mTORC1/PDCD4 pathway. PDCD4 (programmed cell death protein 4), a known tumor suppressor, was further found to have prognostic value in clinical PCa samples. Taken together, our findings suggest that *EPCART* is a cytoplasmic lncRNA, which participates in the modulation of translation in PCa cells.

## Materials and methods

### Cell lines and clinical samples

The prostate cancer cell line LNCaP was obtained from American Type Cell Collection (ATCC, Manassas, VA, USA) and DuCaP was kindly provided by Dr Jack Schalken (Radboud University Nijmegen Medical Center, Nijmegen, the Netherlands). LNCaP cells with deletion of the promoter and 1st and 2nd exon of *EPCART* (*EPCART*-del; clones del-4 and del-56) and their wild-type control (WT) were previously established by our group [11]. To construct the *EPCART* overexpression plasmid, the sequence for the *EPCART* transcript (exons 2–5) was synthesized with additional restriction sites (*Nhe*I and *Xho*I) and then added to the pcDNA3.1(+) plasmid (Invitrogen) by GenScript (Piscataway, New Jersey, USA). Either the pcDNA3.1(+) empty expression vector (Invitrogen) or pcDNA3.1(+) containing *EPCART* was transfected into LNCaP cells with Lipofectamine 3000 transfection reagent (Invitrogen) according to the manufacturer’s instructions. A stable cell pool was selected with 400 μg/ml geneticin (G418; Invitrogen) for several weeks, after which a lower geneticin concentration (200 μg/ml) was used for stable cell line maintenance. *EPCART* expression was determined by quantitative reverse transcription PCR (qRT-PCR). All cell lines were cultured as recommended by the suppliers and tested for mycoplasma contamination regularly.

A formalin-fixed paraffin-embedded (FFPE) tissue specimen of an untreated primary PCa (*n* = 1) for RNA in situ hybridization studies, fresh-frozen tissue samples of untreated primary PCas (*n* = 2) for RACE, and 171 prostate tissue microarray (TMA) samples of untreated primary PCas (*n* = 111) and locally recurrent CRPCs (*n* = 60) for IHC analysis were obtained from Tampere University Hospital (Tampere, Finland).

### Data acquisition and analysis

RNA-seq data curated from different cancer and tissue types were retrieved from MiTranscriptome catalog [8]. RNA-seq data from TCGA-PRAD samples [15] were retrieved from the Genomic Data Commons Data Portal (https://portal.gdc.cancer.gov/) and analyzed as previously described [11]. Clinical data and protein array data from TCGA-PRAD samples were retrieved from cBioPortal (https://www.cbioportal.org/) [16–18]. For PDCD4 expression analyses, Taylor *et al*. [19] whole transcript expression data for human primary and metastatic PCa samples were retrieved from GSE21034, proteome quantification data for primary PCa and localized CRPC (called Tampere PC cohort) from Latonen et al. [20]. RNA-seq normalized expression data for the same samples from Annala et al. [21], and whole proteome quantification data for primary PCa and metastatic CRPC from Iglesias-Gato et al. [22].

### qRT-PCR analysis

RNA was extracted from *EPCART*-del and WT cells with TRIzol Reagent (Invitrogen) according to the manufacturer’s instructions. RNA was converted to cDNA by random hexamer primers (Thermo Scientific) and Maxima reverse transcriptase (Thermo Scientific) following the manufacturers’ instructions. Quantitative PCR was performed by either CFX Opus 96, CFX96, or CFX384 real-time PCR detection system (Bio-Rad). Primer sequences are listed in Supplementary Table 1.

Relative expression values were calculated from quantification cycle (Cq) values, and the target gene measurements were normalized to reference gene (e.g., TBP) values and averaged. Relative gene expression changes were calculated using the 2^−ΔΔCq^ method. 2^ΔCq^ values were used to calculate the significance between each pair (e.g., deletion clone vs. WT).

### 5′ and 3′ end determination

RACE was performed using the SMARTer RACE 5′/3′ Kit (Takara Bio) according to the manufacturer’s instructions. RNA from two fresh-frozen primary PC tissue samples was extracted as previously described [9]. RACE PCR products were obtained using the supplied primers and the appropriate gene-specific primers listed in Supplementary Table 1 and separated on a 1.2% agarose gel. Different sized gel products were extracted with NucleoSpin Gel and PCR Clean-Up Kit (Macherey-Nagel). 5′ RACE products were cloned into pRACE vectors by In-Fhusion HD cloning kit (Takara Bio) and purified by NucleoSpin Plasmid Mini kit (Macherey-Nagel) following manufacturers’ instructions. Purified 5′ RACE-vectors and 3′ RACE PCR-products were sequenced bidirectionally by Sanger sequencing using kit’s universal, gene-specific, or M13 primers (Supplementary Table 1). The Sanger sequencing was performed using BigDye® Terminator v3.1 Cycle Sequencing Kit (Applied Biosystems) and 3500xL Genetic Analyzer (Applied Biosystems) according to the manufacturer’s instructions.

### RNA in situ hybridization

*EPCART* localization in PCa tissues was studied by RNA in situ hybridization. FFPE tissue sections were treated using ViewRNA™ ISH Tissue 2-Plex Assay (Affymetrix) according to the manufacturer’s instructions for a 1-plex assay, as only Fast Red was used for detection. First, slides were briefly deparaffinized in xylene and dehydrated in 100% ethanol. Sections were then pretreated and boiled, and a target probe for *EPCART* (VA1-19503, Affymetrix) and signal amplifiers were hybridized using the ThermoBrite System (Leica Biosystems). A probe for human housekeeping genes (*GAPDH* (glyceraldehyde 3-phosphate dehydrogenase), *ACTB* (actin beta), and *PPIB* (peptidyl-prolyl cis-trans isomerase B); VA1-15726, Affymetrix) was used as a positive control, and a probe for dihydrodipicolinate reductase (*dapB*; VF1-11712, Affymetrix) was used as a negative control in every assay. Signal detection was performed using Fast Red substrate. Slides were counterstained with Gill’s hematoxylin (Sigma-Aldrich). Finally, slides were mounted, first with ImmunoHistoMount (Sigma-Aldrich), and secondly with organic mounting medium. Slides were scanned with Aperio ScanScope XT scanner (Aperio Technologies, Inc.), and imaged at a higher resolution under LSM780 Laser Scanning Confocal Microscope (Zeiss).

### Cellular fractionation

*EPCART* localization was studied in subcellular fractions in LNCaP and DuCaP cells. Nuclear and cytoplasmic RNA was extracted with SurePrep Nuclear or Cytoplasmic RNA Purification Kit (Fisher BioReagents) following the manufacturer’s instructions. Expression of *EPCART*, cytoplasmic control (*GAPDH*), and nuclear control (*U1*) were analyzed by qRT-PCR. Primer sequences are listed in Supplementary Table 1. RNA content in subcellular fractions was calculated as % of transcript abundance = ${2}^{[{\rm{Cq}}({\rm{total\; RNA}})+{\rm{Cq}}({\rm{RNA\; fraction}})]}\times 100$, where total RNA abundance is a sum of nuclear and cytoplasmic fractions.

### RNA-sequencing

For RNA-seq of *EPCART*-deleted and WT clones, three biological replicates were used. Cells were grown for 48 h in a normal medium. RNA was isolated using Trizol (Invitrogen, Thermo Fisher Scientific), treated with RNase-free DNase set (Qiagen), and purified by Monarch RNA Cleanup Kit (New England Biolabs) according to manufacturers’ protocols. The purified RNA was quantified by Qubit 4 Fluorometer (Invitrogen, Thermo Fisher Scientific) and Qubit RNA Broad Range Assay Kit (Invitrogen, Thermo Fisher Scientific), and its purity was assessed by the 260 nm/280 nm ratio. RNA integrity was checked using the 5300 Fragment Analyzer System (Agilent Technologies).

Library preparation was performed using standard polyA enrichment and strand-specific library protocol. Sequencing was performed with a Novaseq6000 (Illumina) in Novogene (Hong Kong, China) for 150 bp paired-­end reads. On average, 105 million cleaned reads per sample were obtained in strand-specific RNA-seq.

### RNA-seq alignment, expression quantification, and differential expression analysis

Read quality of strand-specific RNA-seq data from *EPCART*-del and WT samples were assessed with FastQC v0.11.8 (https://www.bioinformatics.babraham.ac.uk/projects/fastqc/) and reads were aligned to GRCh38 using STAR v2.71a [23] followed by indexing with Samtools v1.8 [24]. Read counts of protein-coding transcripts were calculated using BEDTools v. 2.27.1 sub-command multicov [25] and GENCODE v.38 annotation was used for gene calls. For visualization and clustering of the RNA-seq data, we used variance stabilizing transformation counts calculated by DESeq2 (version 1.22.2) [26], and PCA plots were generated. Differential expression analysis between *EPCART*-del and WT clones was performed using the DESeq2 R package (version 1.22.2), where *p*-values were attained by the Wald test and corrected for multiple testing using the Benjamini and Hochberg method [26].

### Pathway analysis for RNA-seq data

Differentially expressed protein-coding genes (*p* < 0.05) from RNA-seq data of *EPCART*-del and WT clones were analyzed by Ingenuity Pathway Analysis (Qiagen). Canonical Pathways were filtered only to show Signaling Pathways for further analysis for each sample pair (del-4 vs. WT or del-56 vs. WT). Comparison Analysis of del-4 vs. WT and del-56 vs. WT was performed for functions and diseases; only molecular and cellular functions were filtered to be shown for further analysis. IPA uses the *p*-value of overlap, calculated using the right-tailed Fisher’s exact test, to identify significant pathways. The overall activation/inhibition states of Canonical Pathways are predicted based on a *z*-score algorithm. *Z*-scores that are greater than or equal to 2 represent predictions of activation, while predictions of inhibition are made for *z*-scores less than or equal to −2. Log *p-*values > 1.3 (=*p* < 0.05) are considered as significant.

### RNA stability assay

Experiments were performed in biological triplicates. Cells from WT clones were pretreated for 2 h with CHX (at 100 µg/ml) prior to the addition of ActD (at 5 µg/ml) to block transcription. Control cells were treated identically, except that no CHX was added. Samples were taken at 0 and 6 hours of ActD treatment, and the latter was normalized to the former. As there is no transcription in the presence of ActD, the decrease in RNA level between 0 and 6 h is indicative of the degradation rate of that RNA. RNA extraction, cDNA synthesis, and qRT-PCR were carried out as above. Relative gene expression changes were calculated using the 2^−ΔΔCq^ method and *GAPDH* as a reference gene.

### Western blot

Protein samples from cell lysates were prepared as previously described [11]. Proteins were separated by sodium dodecyl sulfate-polyacrylamide gel electrophoresis (SDS-PAGE) on Mini-PROTEAN TGX Precast Protein Gels (Bio-Rad) and transferred to PVDF membrane (Immobilon-P; Milli-pore). Primary antibodies against target proteins (Supplementary Table 2) were used and detected by anti-mouse HRP-conjugated antibodies produced in rabbit (dilution 1:3000; DAKO) or by anti-rabbit HRP-conjugated antibodies produced in swine (dilution 1:5000; DAKO) and Clarity Western ECL Substrate (Bio-Rad) or SuperSignal West Femto Maximum Sensitivity Substrate (Thermo Scientific) with ChemiDoc MP Imaging System (Bio-Rad).

Protein bands were quantified with ImageJ by calculating the relative amounts as a ratio of each protein band relative to the lane’s loading control. Relative protein levels were calculated as fold changes for each pair (e.g., deletion clone/WT) and used for graphs. Quantified ratios were used to calculate the significance between each pair (e.g., deletion clone vs. WT).

### Polysome profiling

Three biological replicates of *EPCART*-del and WT clones were lysed separately for polysome profiling. Each cell lysate was prepared from two 90% confluent 150 mm plates, according to McGlincy et al. [27] with minor changes. Briefly, the complete cell growth medium was changed 2 h prior to harvesting and placed back in a +37 °C/CO_2_ incubator. Then, plates with cells were placed on ice and washed with 8 mL of ice-cold PBS supplemented with 100 µg/mL CHX. PBS was removed and plates were floated in liquid N_2_ to snap-freeze cells. While still frozen, 400 µL of freshly prepared lysis buffer (20 mM Tris, pH 7.4; 150 mM NaCl, 10 mM MgCl_2_, 1% Triton X-100, 1 mM dithiothreitol (DTT), 10 U/mL DNaseI, 100 µg/mL CHX) was dripped onto each plate. Cells were scraped from plates and let slowly melt on ice. Lysates were triturated 10 times through a 26 G needle and clarified by centrifugation for 10 min at 10,000×*g*, +4 °C. The RNA concentration in lysates was measured by a Qubit Broad Range kit (Thermo Fisher Scientific).

In total, 150 µg of lysate was layered onto 10–50% sucrose gradient prepared in polysome buffer (20 mM Tris, pH 7.4; 150 mM NaCl, 10 mM MgCl_2_, 1 mM DTT, 100 µg/mL CHX) and centrifuged at 35 000 rpm (209627.4 × *g*) for 3 h, +4 °C in TH641 rotor (Sorvall). Gradients were fractionated into 15 × 750 µL fractions using an automated piston fractionator (Biocomp) with dual-wavelength *A*_260_/*A*_280_ detection flow cell.

RNA was extracted from polysome fractions with TRIzol LS Reagent (Invitrogen) according to the manufacturer’s instructions with the following changes: 150 µL of chloroform was used; phase separation was performed at 14,000 × *g* for 10 min; 15 µg of GlycoBlue Coprecipitant (Invitrogen) was added to aqueous phase; RNA precipitation was performed at 18,000 × *g* for 30 min. The same volume of RNA solution from each fraction was used for cDNA synthesis. cDNA synthesis and qRT-PCR were carried out as described above. The arithmetic mean of Cq values was calculated for the three technical replicates of each sample, and the RNA percentage for each fraction was calculated as ${ \% {\rm{RNA}}=2}^{{-{\rm{Cq}}}_{x}}/{(2}^{{-{\rm{Cq}}}_{1}}+{2}^{{-{\rm{Cq}}}_{2}}+\ldots +{2}^{{-{\rm{Cq}}}_{y}})\times 100$, where *x* = the number for the fraction that is calculated and *y* = the number for the total number of fractions.

### Sample preparation for mass spectrometry

Five replicate samples from each clone (WT, del-4, del-56) were prepared for MS analysis. Cell pellets (~1 × 10^6^ cells/sample) brought up in cold RIPA lysis buffer with 1% Halt protease inhibitor cocktail (Thermo Scientific) were lysed using ultrasonication for 5 min and incubated for 25 min on ice. The clear supernatant of the cell lysate was collected by centrifugation, avoiding the cell debris, and the total protein concentrations were measured with Bio-Rad DC Protein Assay (Bio-Rad). In total, 50 µg of protein were precipitated with cold acetone, and the precipitate was collected by centrifugation and dissolved in 2% SDS (Sigma-Aldrich) in 50 mM triethylammonium bicarbonate (TEAB) (Honeywell). Protein cysteine disulfide bond reduction was performed with a reducing agent to a final concentration of 3 mM tris-(2-carboxyethyl)-phosphine (Sigma-Aldrich), incubating for 1 h at +60 °C. Samples were transferred to 30 kDa molecular weight cut-off filters (Pall Laboratory), flushed with 8 M urea in 50 mM Tris-HCl (Sigma-Aldrich), and subsequent alkylation of the free reduced cysteine thiols was performed by incubation in dark for 20 min to a final concentration of 50 mM iodoacetamide (Sigma-Aldrich). The protein samples were rinsed multiple times with aliquots of 8 M urea buffer and 50 mM TEAB after which, TPCK-treated trypsin (Sciex; trypsin to protein ratio 1:25) was used to digest the proteins for 16 h at +37 °C. After multiple rinses with aliquots of 50 mM TEAB, peptides were eluted from the filter with 0.5 M sodium chloride (Sigma-Aldrich) and dried in a vacuum centrifuge. The peptide samples were reconstituted in 0.1% trifluoroacetic acid (TFA) and cleaned and desalted with C18 tips (Thermo Scientific). Tips were washed with 2.5% acetonitrile (ACN), 0.1% trifluoroacetic acid and the peptides were eluted from the tips with 80% ACN, 0.1% formic acid (FA) and dried in vacuum centrifuge to be stored for future use. For the MS analysis, the peptide samples were resuspended in 2% ACN, 0.1% FA to a 1.5 µg/µL concentration.

### Mass spectrometry analysis, protein identification, and quantification

Quadrupole time-of-flight mass spectrometer TripleTOF5600 (AB Sciex) coupled to an Eksigent 425 Nano LC system and an Eksigent nano flex cHiPLC system, with Nanospray III electrospray interface (AB Sciex) was used for analysis. 3 µg of the peptide sample was loaded onto a trap column (cHiPLC® ChromXP C18-CL, 3 µm particle size, 120 Å, 75 µm i.d × 5 mm) and loading and desalting were carried out with loading solvent: 2% ACN and 0.1% FA at a 2 µl/min flow rate for 10 min. Consecutively, the trap column was switched to be in line with the reversed phased analytical nano cHiPLC column (cHiPLC® ChromXP C18-CL, 3 µm particle size, 120 Å, 75 µm i.d × 15 cm). The peptide separation was performed using a 120-min gradient of mobile phases A and B, where A is 0.1% FA, 1% ACN in water and B is 0.1% FA in ACN at a 300 nl/min flow rate. The eluted peptides were electro-sprayed into the mass spectrometer via a fused silica emitter (New Objective).

Data dependent acquisition (DDA) method was implemented to generate MS data used to create a spectral library. All 15 samples were used to generate this spectral library containing 2,47,249 spectra, 25,144 peptides from 2519 proteins (at FDR 1%) by searching against the Swiss-Prot human database (canonical 20,370 genes) in the Protein Pilot software® 4.5 (AB Sciex). All 15 samples (two runs/sample) were then rerun again on the same instrument using the same LC conditions, with a data-independent (SWATH) acquisition mode to acquire protein quantification data. Retention time normalization was carried out using 6 peptides, each of the two highest-score proteins (HSPD1, HSPA8). Altogether 2083 proteins were quantified (at FDR 1%) for each sample processing against the spectral library using the PeakView® (AB Sciex) and MarkerView® software.

### Immunohistochemical analysis

PDCD4 protein expression levels in prostate carcinomas were validated by IHC analysis from FFPE TMA samples. IHC staining of PDCD4 was performed by Ventana BenchMark GX IHC/ISH system (Ventana Medical Systems, Roche), ultraView Universal Dab Detection Kit (Roche), and anti-PDCD4 antibody (EPR3431, Abcam) in 1:4000 dilution. Slides were scanned with NanoZoomer S60 Digital slide scanner (Hamamatsu Photonics) with a 20× objective.

Nuclear and cytoplasmic staining intensities of PDCD4 were classified on a scale from 0 to 3 with negative (0), weak (1), moderate (2), or strong (3) staining within cancerous areas. If possible, a minimum of 200 cells were calculated for each sample. The Histoscore (*H*-score) was calculated as *H*-score = (0 × percentage of cells with absent cytoplasmic staining) + (1 × percentage of “1+” cells) + (2 × percentage of “2+” cells) + (3 × percentage of “3+” cells).

### Statistical analyses

Mann–Whitney *U* tests were used to analyze the association between *PTEN*-sample groups in TCGA-PRAD data. Unpaired two-tailed Student’s *t* tests were used to calculate the significance between control and experimental conditions in PCR and immunoblot experiments. *P*-values < 0.05 were considered statistically significant.

Kaplan–Meier survival analysis and log-rank tests for TCGA-PRAD and Taylor et al. data were used to determine the progression-free survival between samples divided by their first quartile expression. For correlation analysis in Tampere PCa and TCGA-PRAD cohorts, Spearman’s rank correlation coefficient was calculated for *EPCART* and PDCD4 expressions in a pairwise manner.

The protein quantification data from *EPCART*-del and WT clones were log_2_-transformed, and the replicate MS analyses were combined by taking means. The coefficient of variation (CV) was calculated for samples originating from the same sample type and passage in order to identify and exclude quantified proteins with poor repeatability (CV ≥ 30%). Due to the small number of samples processed, only descriptive analysis and results are reported for individual proteins. These include means by sample types and the associated log_2_ fold changes (log_2_FC) between different sample types. Proteins with log_2_FC > log_2_(1.5) and <log_2_(0.67) were included in the pathway analyses. R software (v4.1.2, R Core Team) was used to process the data and perform the descriptive analyses.

## Results

### Expression of *EPCART* is elevated in prostate cancer

**Fig. 1:**
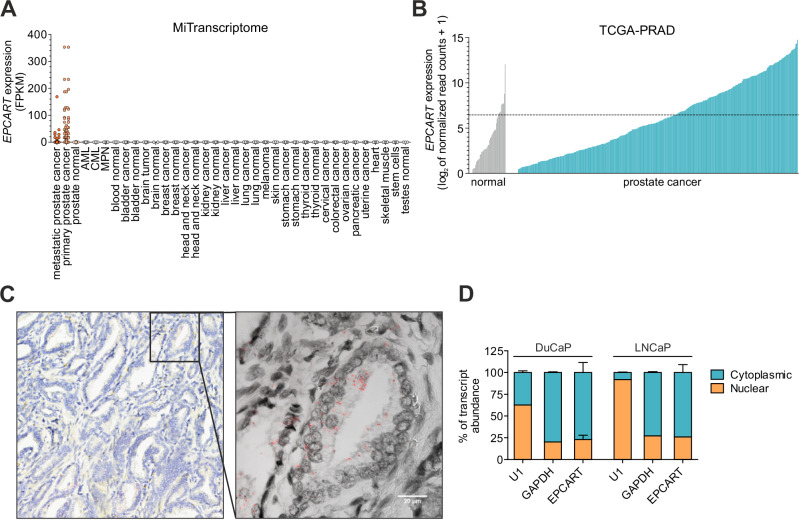
Characterization of *EPCART* expression. **A** *EPCART* expression across human tissue and blood samples in MiTranscriptome lncRNA catalog. **B** *EPCART* expression in adjacent normal prostate and primary prostate cancer samples in TCGA-PRAD dataset. The dashed line represents the overexpression cutoff for *EPCART*. **C** *EPCART* localization in primary PCa tissue according to RNA in situ hybridization assay. The sample slide was imaged with a brightfield slide scanner (left) and a confocal microscope (right) for higher resolution. Signals from *EPCART* transcripts were detected with Fast Red and the slides were counterstained with hematoxylin. **D** Expression of *EPCART* in cytoplasmic and nuclear fractions of two different PCa cell lines. Subcellular RNA was extracted from cells grown in a normal medium for 3 days and analyzed by qRT-PCR. *U1* was used as a nuclear marker and *GAPDH* as a cytoplasmic marker.

We previously identified *EPCART* to be a PCa-associated lncRNA and a potential independent biomarker for PCa progression [9, 11]. To understand *EPCART* expression in PCa in more detail, we studied multiple publicly available transcriptome sequencing datasets. *EPCART* was verified to be expressed in a PCa-specific manner in MiTranscriptome lncRNA catalog data [8], where *EPCART* showed little or no expression in other tissue types than primary and metastasized PCa (Fig. 1A). *EPCART* was also found to be highly abundant in PCa, as its expression was one of the highest among other differentially expressed prostate-specific lncRNAs (3rd most expressed based on 99th percentile expression and 6th most expressed based on 95th percentile expression; Supplementary Table 3). To further investigate the fraction of PCa tumors where *EPCART* is expressed, we analyzed data from The Cancer Genome Atlas Prostate Adenocarcinoma (TCGA-PRAD) samples. *EPCART* was overexpressed [fold change (FC) > 2, when compared to expression in adjacent normal (median + standard deviation)] in 37% of the PRAD cases in this dataset (Fig. 1B).

Next, we wanted to validate the transcript structure of *EPCART* and study its possible variants in PCa. Based on our previous RNA-sequencing (RNA-seq) data from PCa tumor samples, a majority of the transcripts have four exons [9], although we detected a short alternative exon at the beginning of the 5′ end in some of the RNA-seq samples (Supplementary Fig. 1A). To verify the exon structure of the transcripts, we performed Sanger sequencing to the exon-exon boundaries in a PCa tumor sample with high *EPCART* expression. With this, exon-exon boundaries for five exons were confirmed (Supplementary Fig. 1B). To determine the ends of the *EPCART* transcript, we used 5′ and 3′ rapid amplification of cDNA ends (RACE) technique. While the 3′ end of the *EPCART* transcript showed consistency (Supplementary Fig. 1C), the 5′ end had prominent variation (Supplementary Fig. 1D). We observed sequences from the intronic area between exon 1 and 2 for the majority of the 5′ RACE products (62/99), and only a small portion of the sequences (3/99) contained exons 1 and 2 without the intron (data not shown).

Some lncRNAs have been found to encode micropeptides from their short open reading frames (ORFs) [28] and to have a role in cancer development [29, 30]. To assess if *EPCART* transcript has any ORFs, we analyzed its coding potential with Coding Potential Calculator 2.0 [31]. The analysis found a short 74 aa long hypothetical peptide with low coding probability (Supplementary Table 4). In some cases, the micropeptide sequences can share homology with full-length proteins and have a function through molecular similarity. For example, lncRNA LINC00689 encodes for a 50aa peptide that exhibits molecular mimicry of the SRP19 (signal recognition particle 19) protein [32]. However, BLAST homology search with a hypothetical amino acid sequence of *EPCART* did not find any homologous proteins, nor was it found to be in any well-known protein families in InterPro search (https://www.ebi.ac.uk/interpro/). Together, these results suggest that there is a low probability for *EPCART* to produce a functional micropeptide.

To understand where *EPCART* is located in PCa cells, we performed an RNA in situ hybridization assay in a PCa tumor specimen with high *EPCART* levels. The assay confirmed *EPCART* transcripts to be expressed in PCa tumor cells (Fig. 1C, Supplementary Fig. 2A–C). *EPCART* was mostly located in the cytoplasm of the cells (Fig. 1C, Supplementary Fig. 2C), which was also confirmed by RNA subcellular localization studies in vitro assessed by nuclear-cytoplasmic fractionation (Fig. 1D).

### *EPCART* silencing induces inhibition of translation

We have previously created *EPCART* knock-out cells in the LNCaP prostate cancer cell line and found that lack of *EPCART* decreased cell proliferation and migration [11]. The advantageous impact of *EPCART* on cell proliferation was further validated in LNCaP cells that were stably overexpressing *EPCART* (Supplementary Fig. 3A, B). To understand the mechanisms behind this phenotype, we performed RNA-seq for the *EPCART*-deletion (*EPCART*-del) clones (del-4 and del-56) and the wild-type (WT) clone. To evaluate the similarity of *EPCART*-del clones, we assessed their expression profiles; both principal component analysis and the comparison of all the significant log_2_FC values (p-value < 0.05) of protein-coding genes revealed that while there were some differences between the *EPCART*-del clones, their expression profiles were mostly similar (Supplementary Fig. 4A, B). We found 367 and 560 protein-coding genes to be significantly up- and downregulated (log_2_FC < −1 and >1, *p-*value < 0.05), respectively, in the del-4 clone, and 437 and 523, respectively, in the del-56 clone when compared to WT clone (Supplementary Table 5). Of these, 156 upregulated and 280 downregulated genes were common between the two *EPCART*-del clones (Supplementary Fig. 4C).

**Fig. 2:**
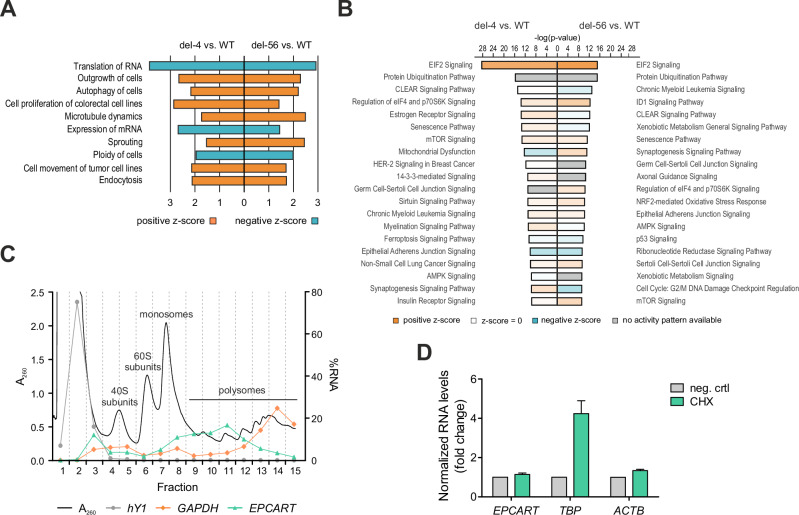
*EPCART* silencing causes translational inhibition. **A** Identification of the molecular and cellular functions in *EPCART*-del clones based on RNA-seq data. Top 10 most significant (*z*-score > 2) functions shared by both clones are shown. **B** Canonical signaling pathway analysis of *EPCART*-del clones. Pathways were ordered based on their *p*-values, and the top 25 pathways with the smallest *p-*values are shown. The intensity of the color represents the strength of the z-score (the stronger the color, the higher the score); individual scores are listed in Supplementary Table 7. Right-tailed Fisher’s exact test was used for evaluating *p-*values. **C** RNA percentage of *EPCART* in polysome profiling fractions of WT clones (LNCaP). RNA was extracted from each fraction and analyzed by qRT-PCR. %RNA of each fraction was calculated for *EPCART*, *GAPDH* (positive control), and *hY1* (negative control), and plotted in the same graph with a representative polysome profile (*A*_260_). Profile peaks that correspond to ribosomal subunits, monosomes, and polysomes are indicated on the graph. The graph presents one replicate (#3). **D** Changes in *EPCART* stability in response to ribosome stalling. *EPCART* WT cells (LNCaP) were treated with and without CHX, which blocks the translation. Control and treated samples were then taken at 0 and 6 h after ActD addition, which blocks transcription, and transcript levels were quantified by qRT-PCR to assess the degradation rate of RNAs. Bars show the mean fold change of three biological replicates of 6 h samples normalized to 0 h control samples. Treated samples were further normalized to control (untreated) samples. Error bars, SD of three biological replicates.

Next, we performed pathway analysis (IPA) using all significantly up- or downregulated genes identified in RNA-seq (*p-*value < 0.05). The most affected class of molecular and cellular functions was RNA translation, which was inactivated in both *EPCART*-del clones, with further functions related to mitosis, cell proliferation, and cell death (Fig. 2A, Supplementary Table 6). Analysis of canonical signaling pathways showed significant activation (*z*-score > 2) of eIF2 signaling, a pathway related to protein synthesis [33], as well as major changes (*p-*value < 0.0001) in other translation-associated pathways, including protein ubiquitination, eIF4, and p70S6K signaling, and mTOR signaling pathways (Fig. 2B, Supplementary Table 7).

Since *EPCART* silencing affected translation pathways and because *EPCART* is localized to the cytoplasm (Fig. 1C, D), we tested whether *EPCART* is directly bound to ribosomes, the site of translation. For this, we assessed the abundance of *EPCART* transcripts in different fractions collected from polysome profiling in WT clone (LNCaP). *EPCART* was indeed detected mostly in polysomal fractions (Fig. 2C, Supplementary Fig. 5A), and similar to other polysome-associated lncRNA, especially in the more “light” polysomal fractions that contain only a few ribosomes [34]. Previous studies have suggested that ribosomes may play a role in the degradation of ribosome-associated lncRNAs [34, 35]. To investigate if *EPCART* stability is affected by polysomal binding, we stalled the ribosomal elongation by cycloheximide (CHX) inhibited the transcription by Actinomycin D (ActD) in vitro and measured the degradation rate of transcripts by qRT-PCR (Fig. 2D). The expression of *EPCART* was not significantly affected by the treatments, in contrast and similarly to what has been previously published for TATA-box binding protein gene (*TBP*) [34]. This indicates that degradation may not be the main reason for *EPCART* to bind ribosomes. To further assess if the global translation activity is greatly affected by *EPCART* silencing, we performed polysome profiling in *EPCART*-del and WT clones. No significant changes were detected in the polysome gradient profiles between the samples (Supplementary Fig. 5B). Collectively, these results indicate that the effects of *EPCART* on translation likely depend more on regulating specific populations of genes through translation-associated pathways than direct ribosome associations of *EPCART* itself.

### *EPCART* modulates downregulation of PDCD4 through AKT/mTOR pathway

**Fig. 3:**
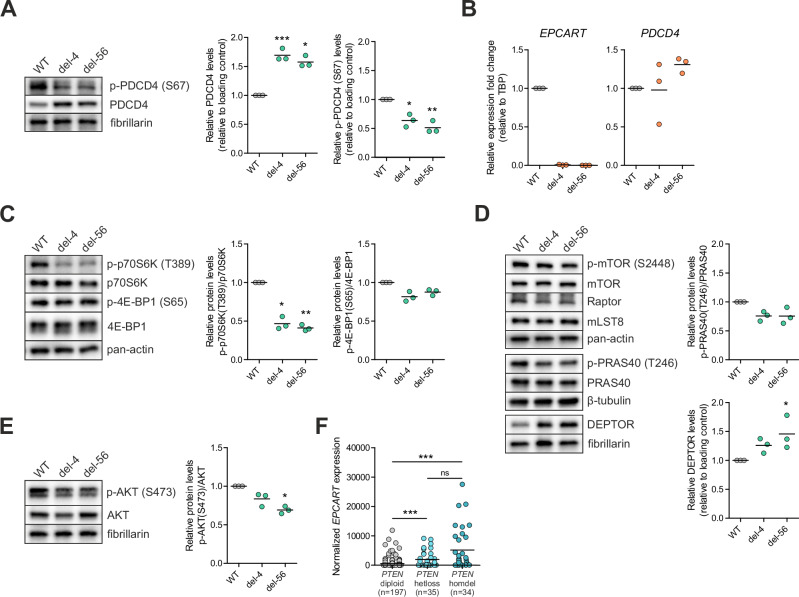
Translation inhibition works through the AKT/mTOR/PDCD4 pathway in *EPCART*-del cells. **A** Immunoblot analysis of PDCD4 and its phosphorylation in *EPCART*-del and WT clones. Fibrillarin was used as a loading control. Intensities for each band were quantified in three biological replicates and their relative values were plotted. Quantified ratios (target protein/loading control) of each pair (deletion clone vs. WT) were assessed with an unpaired two-tailed *t*-test. **B** RNA expression analysis of *EPCART* and *PDCD4* in *EPCART*-del and WT clones. Expression was analyzed by qRT-PCR in three biological replicates using *TBP* as a reference gene. Relative expression values (2^−^^ΔΔCq^) for three biological replicates were plotted. 2^ΔCq^ values of each pair (deletion clone vs. WT) were assessed with an unpaired two-tailed *t*-test. **C** Immunoblot analysis of p70S6K, 4E-BP1, and their phosphorylated forms in *EPCART*-del and WT clones. Pan-actin was used as a loading control. Intensities for each band were quantified in three biological replicates and their relative values were plotted. Quantified ratios (target protein/loading control) of each pair (deletion clone vs. WT) were assessed with an unpaired two-tailed *t*-test. **D** Immunoblot analysis of mTORC1 components and their phosphorylated forms in *EPCART*-del and WT clones. Pan-actin, β-tubulin, or fibrillarin were used as a loading control. Intensities for each band were quantified in three biological replicates and their relative values were plotted. Quantified ratios (target protein/loading control) of each pair (deletion clone vs. WT) were assessed with an unpaired two-tailed *t*-test. **E** Immunoblot analysis of AKT and its phosphorylated form in *EPCART*-del and WT clones. Pan-actin was used as a loading control. Intensities for each band were quantified in three biological replicates and their relative values were plotted. Quantified ratios (target protein/loading control) of each pair (deletion clone vs. WT) were assessed with an unpaired two-tailed *t*-test. **F** *EPCART* expression in PCa tumors with *PTEN* deletion according to TCGA-PRAD data. Samples were divided by their *PTEN* status [15]: normal diploid, loss of heterozygosity (hetloss), or homologous deletion (homdel). The significance of expression differences was assessed with a nonparametric Mann–Whitney test. In **A**–**F**, the arithmetic mean expression of each group is marked with a black line. *p < 0.05; **p < 0.01; ***p < 0.001.

To gain insight into the effect of *EPCART* silencing on translated proteins, we performed a quantitative proteomics assay by sequential window acquisition of all theoretical fragment ion spectra mass spectrometry (SWATH-MS) for *EPCART*-del and WT clones. These experiments revealed 23 proteins that were differentially expressed (log_2_ FC < −0.5 or >0.5) in the two deletion clones when compared to the WT clone (Table 1, Supplementary Table 8). Interestingly, the most significantly overexpressed protein was PDCD4, which is known to bind to translation initiation factor eIF4A1 and inhibit its function by preventing RNA binding [36]. The overexpression of PDCD4 in *EPCART*-del clones was validated by immunoblotting (Fig. 3A). At the RNA level, overexpression of *PDCD4* was not evident (Fig. 3B), suggesting that *EPCART* is associated with the stability of PDCD4 protein.

**Table 1:** Differential protein expression results in *EPCART*-knockout (LNCaP) cells.

Gene symbol	Differences (log2FC)	
	del-4 vs. WT	del-56 vs. WT
PDCD4	1.29	1.24
PTGR1	1.26	0.69
CBX5	0.87	0.62
OTUD6B	0.93	0.7
GSTM3	0.77	0.81
AGPS	0.68	1.3
DHRS7	0.67	0.75
AHCYL1	0.66	0.67
FKBP1A	0.62	0.73
SNX3	0.62	0.8
MAPK1	−0.59	−0.61
MARS2	−0.59	−0.72
FOLH1	−0.61	−0.81
PDXDC1	−0.62	−0.61
GPD2	−0.74	−0.71
CTSC	−0.78	−0.9
SLC9A3R2	−0.83	−0.6
ATP1B1	−0.88	−1.1
NCAM2	−0.89	−0.93
VTA1	−0.9	−0.88
LDHB	−0.94	−1.39
SCCPDH	−0.97	−0.63
AGR2	−1.25	−1.01

Genes that were overexpressed (log2FC > 0.5) or downregulated (log2FC < −0.5) in both *EPCART*-KO clones are shown.

PDCD4 is downstream of the mechanistic target of the rapamycin complex 1 (mTORC1) signaling pathway that functions in the control of general translation [37]. mTORC1 activates p70 S6 kinase (p70S6K) through phosphorylation at Thr389 [38]. Subsequently, PDCD4 is phosphorylated by p70S6K at Ser67, which leads to ubiquitination and degradation of PDCD4 [39]. In *EPCART*-del clones, we found p70S6K to be less activated when compared to WT (Fig. 3C), and PDCD4 to be less phosphorylated at Ser67 (Fig. 3A). These results indicate that PDCD4 overexpression in *EPCART*-del clones is due to inactivation of p70S6K that leads to suppression of PDCD4 degradation and that mTORC1 activity is inhibited in *EPCART*-del cells. As mTOR inhibition suppresses mRNA translation [37, 40], this supports the idea of translational inhibition occurring in *EPCART*-del cells.

To better understand the causes of mTORC1 inhibition, we investigated potential changes in either the amount or activity of different protein components of mTORC1 in *EPCART*-deleted cells. The complex consists of mTOR that has the catalytic activity, Raptor (regulatory-associated protein of mTOR) that facilitates substrate binding to mTOR, mLST8 (mammalian lethal with SEC13 protein 8) that stabilizes mTOR, and two inhibitory subunits, PRAS40 (proline-rich Akt substrate of 40 kDa) and DEPTOR (DEP domain-containing mTOR-interacting protein) [38]. No major changes were observed in levels of mTOR, phospho-mTOR Ser2448, Raptor, or mLST8, but we found phosphorylation of PRAS40 at Thr246 decreased and DEPTOR expression level increased in *EPCART*-del clones (Fig. 3D). The main inducer of Thr246 phosphorylation in PRAS40 is AKT (protein kinase B) [41], indicating that the kinase activity of AKT might be decreased in *EPCART*-del cells. Hence, phosphorylation of AKT was assessed by immunoblotting and, indeed, we found phosphorylation of AKT at Ser473 to be reduced in *EPCART*-del clones when compared to the WT clone (Fig. 3E).

In PCa, PI3K/AKT/mTOR signaling is often elevated, predominantly due to PTEN (phosphatase and tensin homolog) loss-of-function, which promotes cell survival [42]. LNCaP cells also have inactive PTEN that leads to increased AKT activation [43]. To investigate if *EPCART* is associated with PI3K/AKT/mTOR signaling in clinical samples, we examined the expression of *EPCART* in TCGA-PRAD samples [15]. More specifically, we investigated *EPCART* expression in samples with either *PTEN* homozygous deletion (homdel), *PTEN* loss of heterozygosity (hetloss), or no deletion (diploid). We found *EPCART* expression to be the highest in *PTEN* homdel samples, although the *EPCART* expression was significantly elevated also in *PTEN* hetloss samples (Fig. 3F). These results further reinforce the interaction between *EPCART* and PI3K/AKT pathway.

### PDCD4 has prognostic potential in prostate cancer

**Fig. 4:**
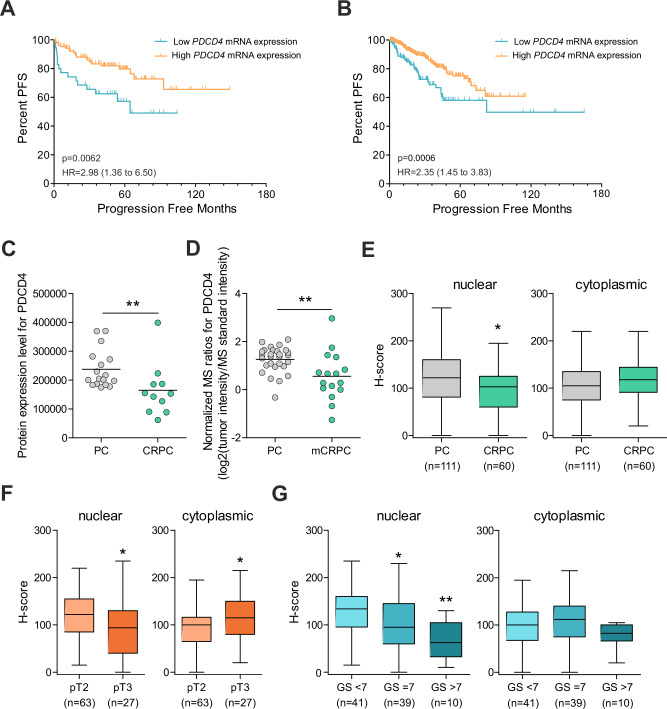
PDCD4 expression is associated with PCa progression. **A**, **B** Kaplan–Meier estimates of progression-free survival (PFS) in PCa patients divided by *PDCD4* mRNA expression in Taylor et al. (**A**) and in TCGA-PRAD cohorts (**B**). The first quartile was used as a cutoff between low and high mRNA expression. *P-*values were calculated by log-rank test. HR, hazard ratio. **C**, **D** PDCD4 protein expression in primary PCa (PC) and in CRPC based on MS results in our Tampere PC cohort (**C**) and Iglesias-Gato et al. cohort (**D**). **E** IHC analysis of PDCD4 in untreated primary PCa (PC) and locally recurrent CRPC samples. *H*-scores for nuclear and cytoplasmic staining are shown separately. **F**, **G** IHC analysis of PDCD4 in untreated primary PCa samples that were divided into groups by pT stage (**F**) or Gleason score (**G**). *H*-scores for nuclear and cytoplasmic staining are shown separately. Error bars display the minimum and maximum values. In **C**–**G**, the arithmetic mean expression of each group is marked with a black horizontal line, and the significance of expression difference to the leftmost sample group was assessed with a nonparametric Mann–Whitney test. *p < 0.05; **p < 0.01; ***p < 0.001.

Since we found PDCD4 stability to be affected by *EPCART* in vitro, we studied whether PDCD4 is associated with *EPCART* also in clinical PCa. For this, we analyzed the effect of *PDCD4* expression in different PCa datasets. We found that low *PDCD4* expression correlated with decreased progression-free survival of PCa in Taylor et al. [19] and TCGA-PRAD [15] cohorts (Fig. 4A, B). PDCD4 protein expression was also found to be decreased in CRPC when compared to primary PCa in two cohorts with locally advanced and distal metastases [20, 22] (Fig. 4C, D), therefore associating low PDCD4 protein levels to more aggressive PCa. Similarly, immunohistochemical (IHC) analysis of PCa specimens revealed PDCD4 levels to decrease from primary PCa to CRPC, but only in the nucleus, whereas cytoplasmic PDCD4 levels were slightly increased (Fig. 4E, Supplementary Fig. 6). Decreased nuclear PDCD4 levels and increased cytoplasmic PDCD4 levels also associated with higher pathological T stage and Gleason score (Fig. 4F, G), indicating that PDCD4 localization has prognostic significance.

Additionally, we found a moderate negative correlation with *EPCART* expression and PDCD4 protein expression in our Tampere primary PCa specimens (Spearman’s *ρ* = −0.38; *n* = 17) [20], but not with *PDCD4* mRNA expression (Spearman’s *ρ* = 0.13; *n* = 28) (Supplementary Table 9), supporting the idea that expression of *EPCART* and PDCD4 protein regulation are linked. Furthermore, a comparison of RNA expression and protein array data in the TCGA-PRAD cohort indicated a negative correlation between PCDC4 and *EPCART* expression (Spearman’s *ρ* = −0.21; *n* = 283).

## Discussion

Prostate cancer is the second most commonly occurring cancer in men worldwide [44]. While conventional treatments are able to cure the great majority of the primary PCs globally, the prognosis for advanced PC is still poor and the current therapies can only prolong the survival time for these patients [45–47]. The behavior of prostate cancer varies notably from patient to patient, creating a clinical challenge and a high demand for molecular markers and targets [48]. One of the factors that plays a significant role in PCa development is lncRNAs [14]. While hundreds of PCa-associated lncRNAs have been found, the function and molecular mechanism are known only for a handful of them. Here, we investigated the role of *EPCART*, a PCa-associated oncogenic lncRNA [11], in PCa cells. By using *EPCART*-deleted PCa cells, we found that *EPCART* promotes mRNA translation, with one of its downstream targets being the translation-inhibitory tumor suppressor PDCD4. This regulation was further identified to take place through the PI3K/AKT/mTORC1 pathway.

In recent years, an increased number of lncRNAs have been recognized to function in the cytoplasm [49, 50]. Growing evidence suggests that some cytoplasmic lncRNAs can act as modulators of translation, either by regulating translational factors or through signaling pathways that control protein translation [50]. We showed here that *EPCART* is a translation-regulating lncRNA. In *EPCART*-deleted cells, we observed downregulation of translation at the mRNA level and *EPCART* was found to localize into polysomal fractions. A majority of the cytoplasmic lncRNAs also appear to associate with actively translating ribosomes [34]. For some of these RNAs, ribosomes might serve as a place of degradation [34], while other cytoplasmic RNAs might be translated into short peptides [28], or be structurally associated with the translational machinery. *EPCART* was not found to possess protein-coding potential, indicating that *EPCART* functions as a transcript. We validated the structure of *EPCART* transcripts and found an alternative exon at its 5′ end, with notable variation at the 5′ end. This suggests *EPCART* to be inefficiently spliced, which is quite common for lncRNAs [51, 52]. Additionally, we did not detect *EPCART* to be degraded after chemical inhibition of translation nor was *EPCART* equally distributed among all the polysomal fractions used as an indication of structural association with ribosomes. *EPCART* was, however, found in “light” polysome fractions, indicating that it could have a role in the early steps of translation. Nevertheless, we could not exclude the possibility that the polysomal binding of *EPCART* is nonspecific, and we found no evidence that the ribosomal association of *EPCART* is the cause for the observed translational effect. Instead, we found *EPCART* to function through the PI3K/AKT/mTOR signaling pathway to indirectly downregulate translation through mediators, including PDCD4.

MTORC1 is one of the central pathways regulating protein translation [53]. Through phosphorylation of its substrates, including p70S6K and 4E-BPs, mTORC1 promotes protein synthesis [53]. Translation regulation is also an essential part of cell growth and proliferation and, therefore, often dysregulated in cancer cells [54]. In *EPCART*-deleted cells, we found the mTORC1/p70S6K pathway to be altered at the protein level. Additionally, we discovered PDCD4, a p70S6K downstream substrate, to be substantially less degraded in *EPCART* knockout cells. PDCD4 has been shown to repress translation initiation through inhibition of eIF4A activity [54], thus indicating a potential mechanism for the observed translational repression after *EPCART* silencing. As the polysome profiling did not indicate major changes in the global translation, the effect *EPCART* has on translation is potentially directed against specific mRNAs. Translational regulation of specific genes through the mTOR pathway is well established [37, 40], and examples of lncRNAs as part of this regulation have been described [50].

To better understand the upstream regulation of mTORC1 inhibition in *EPCART*-deleted clones, we investigated individual mTORC1 partners and their phosphorylation. Both endogenous mTOR inhibitors, PRAS40 and DEPTOR, were dysregulated, DEPTOR by overexpression and PRAS40, which is dissociated from mTORC1 by AKT phosphorylation [53], was dephosphorylated when *EPCART* was silenced. Furthermore, we observed the activity of AKT to be inhibited by dephosphorylation in the *EPCART*-deleted cells. These results suggest that *EPCART* modulates the activity of mTORC2, which is also inhibited by DEPTOR and participates in the activation of AKT via phosphorylation at Ser473 [37]. While the exact mechanism of this regulation is still unclear, one potential target could be AGR2, which was the most downregulated protein in *EPCART*-del cells according to proteomics results and has been associated with mTORC2 in cancer [55]. Additionally, in PCa tumors, high *EPCART* expression was associated with *PTEN* loss, a common aberration in cancer cells that leads to activation of the PI3K/AKT pathway. Together, our findings indicate that *EPCART* modulates protein synthesis via PI3K/AKT/mTORC1/PDCD4 pathway in PCa cells. This pathway is known to promote cell growth and proliferation [53], which makes the inhibition of it a probable explanation for the decreased proliferation of *EPCART* knockout cells [11]. The exact molecular interactions through which *EPCART* affects this pathway remain to be investigated in future studies.

Previously, we and others showed high expression of *EPCART* to be a potential independent prognostic marker in PCa [11, 56]. Here, we further demonstrated *EPCART* expression to be a highly PCa-specific marker when compared to any normal or cancer tissue. Moreover, we found low PDCD4 mRNA and protein expression to be associated with poor prognosis in primary PCa samples. This is consistent with the results in other studies, where PDCD4 downregulation has been shown to be involved in and to be a potential prognostic marker for many solid tumors [57]. Interestingly, we observed decreased nuclear and increased cytoplasmic localization of PDCD4 to associate with more aggressive PCa. While similar differential distribution of PDCD4 between nuclear and cytoplasmic compartments has been observed before in other cancer cells and has clinical significance in a few other cancers [57–59], our results are the first to report the effect in PCa. Additionally, we observed a negative correlation between *EPCART* and PDCD4 in PCa tumor samples, suggesting there to be a subpopulation of PCa tumors, the protein synthesis of which is modulated by *EPCART* through AKT/mTOR/PDCD4 pathway.

To conclude, our studies found *EPCART* to be a cytoplasmic lncRNA that has a functional role in the modulation of protein translation through the PI3K/AKT/mTOR/PDCD4 pathway in PCa. We also provide more evidence of the tumor suppressive role of PDCD4 in PCa tumors in collaboration with *EPCART*. Whether this signaling would lead to a worse PCa prognosis should be studied in larger prospective studies.

## Supplementary information

Supplementary Figures

Supplementary Tables 1-9

## Data Availability

The RNA-seq data generated in this study are publicly available in Gene Expression Omnibus at GSE249960. Raw MS data for this study were generated at Tampere Mass Spectrometry Facility, Tampere University in Tampere, Finland. Derived proteomics data supporting the findings of this study are available from the corresponding author upon request and the most relevant derived data can be found in Supplementary Table 8.
