# Supplementary material for: Long noncoding RNA EPCART regulates translation through PI3K/AKT/mTOR pathway and PDCD4 in prostate cancer
Source: Cancer Gene Ther. 2024 Aug 15;31(10):1536–46. doi: 10.1038/s41417-024-00822-3 (PMC11489079; doi:10.1038/s41417-024-00822-3)
Supplement: Supplementary file 1 — Supplementary Figures [file 41417_2024_822_MOESM1_ESM.pdf]

Supplementary figures

A

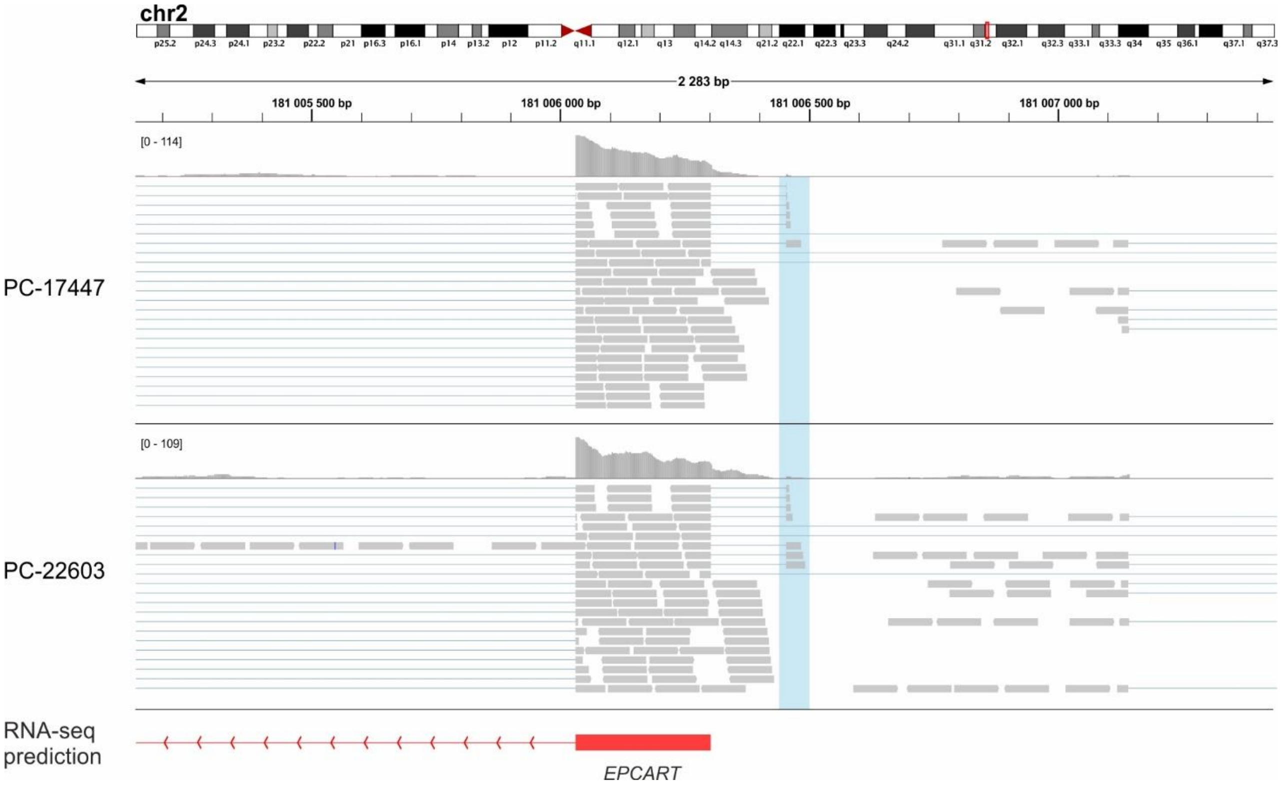

B

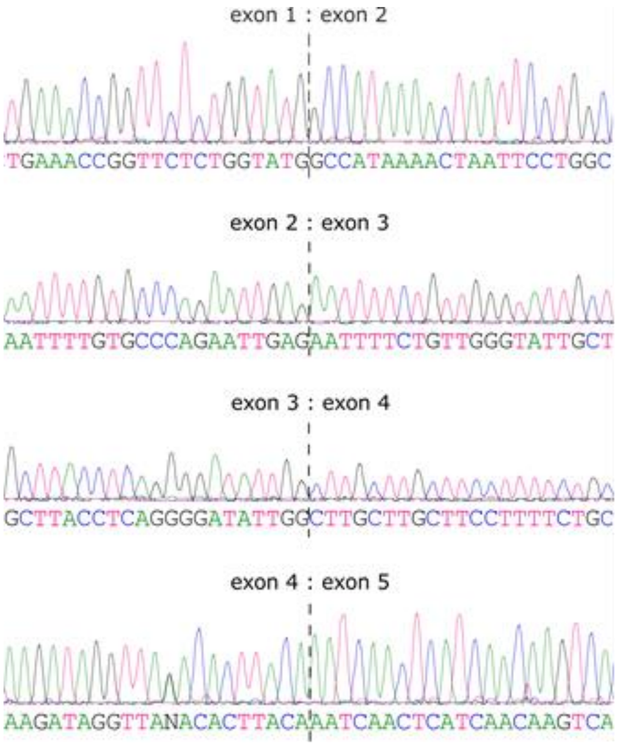

C

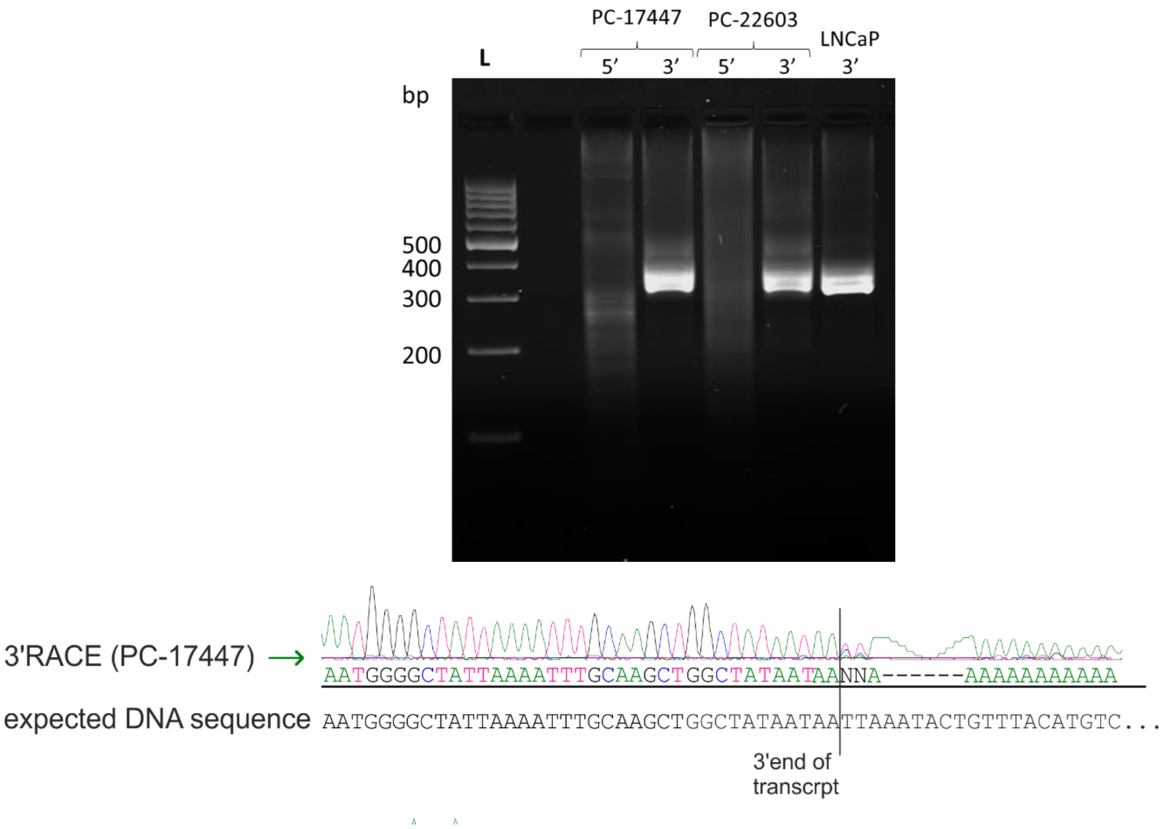

PC-17447
PC-22603
LNCaP
L
5′
3′
5′
3′
3′
bp
500
400
300
200
3'RACE (PC-17447)
AATGGGGCTATTAAAATTTGCAAGCTGGCTATAATAANNA------AAAAAAAAAAA
expected DNA sequence
AATGGGGCTATTAAAATTTGCAAGCTGGCTATAATAATTAAATACTGTTTACATGTC...
3'end of
transcrpt

D

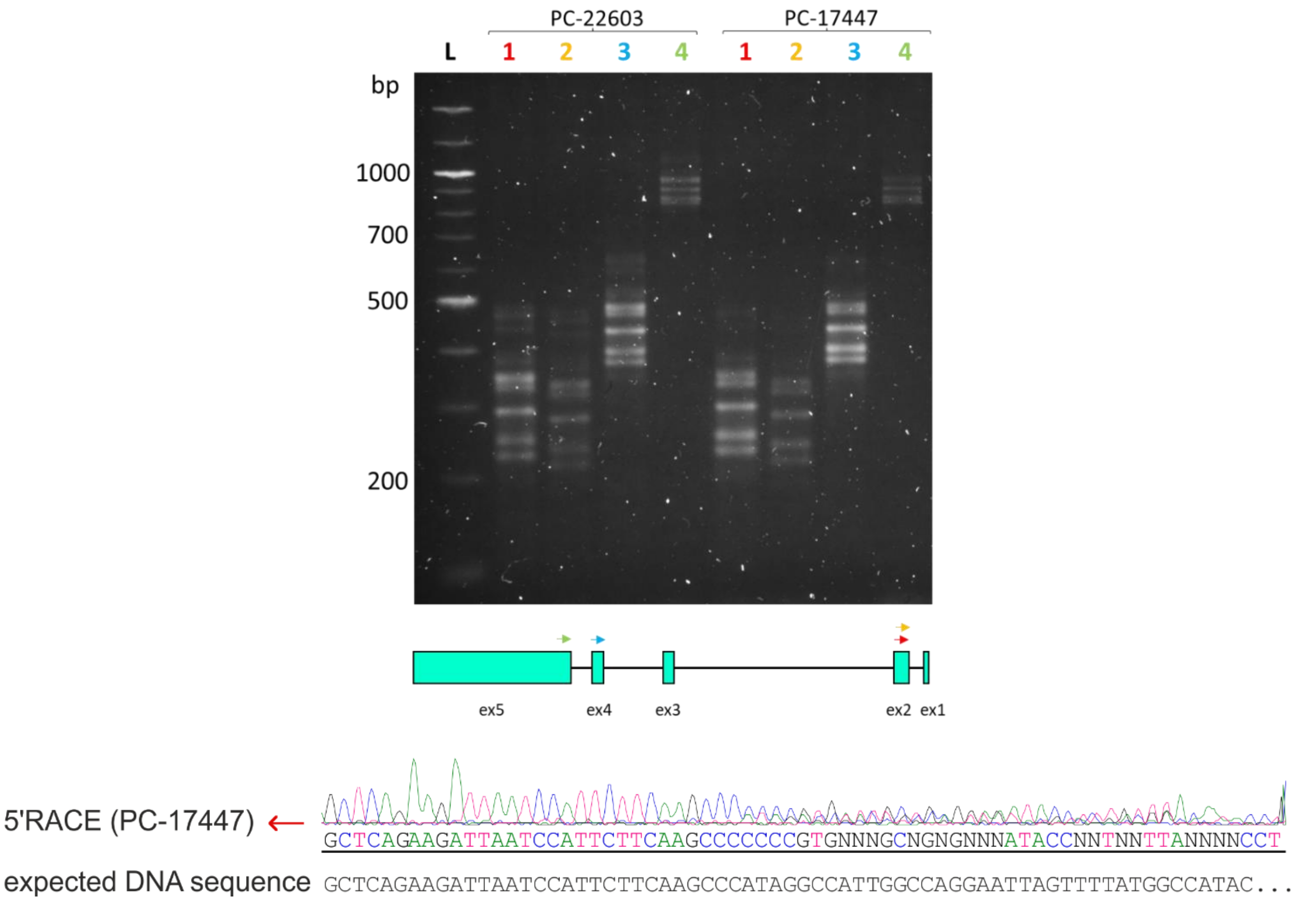

PC-22603
PC-17447
L
1
2
3
4
1
2
3
4
bp
1000
700
500
200
ex5
ex4
ex3
ex2
ex1
5'RACE (PC-17447)
GCTCAGAAGATTAATCCATTCTTCAAGCCCCCCCGTGNNNGCNGNGNNNATACCNNTNNTTANNNNCCT
expected DNA sequence
GCTCAGAAGATTAATCCATTCTTCAAGCCCATAGGCCATTGGCCAGGAATTAGTTTTATGGCCATAC...

Supplementary Figure 1. Characterization of *EPCART* transcript structure. **A**) At the 5' end of *EPCART* a short exon (highlighted as blue) was detected in RNA-seq of PC tissue samples (PC-17447 and PC-22603 are here used as examples) that was not present in all transcripts. The original RNA-seq prediction without the additional exon is marked as red. **B**) Exon boundaries of *EPCART* were validated in a PC tissue sample (PC-17447 or PC-22603) by Sanger sequencing. **C-D**) Rapid amplification of cDNA ends (RACE) was used to determine the 3' (C) and 5' (D) ends of *EPCART* transcript in two PC tumor sample (PC-17447 and PC-22603) and 3' end also in LNCaP cells. RACE products (1st PCR products for 5' RACE in D and 2nd nested PCR products for 3' RACE in C) were extracted from an agarose gel and Sanger sequenced. In C, one strong 3' RACE band was extracted and analyzed. Also 2nd nested PCR products for 5' end are shown, however these products were not used for analysis. Variation was observed in the 5' RACE product sequences (D, below image), which is why all seen bands were extracted and cloned into vectors before sequencing. Arrows before sequences indicate the direction of *EPCART* transcript (5'→3'). L, GeneRuler 100 bp (Thermo Scientific) in C and 100 bp Quick-Load Purple (NEB) in D; 1-4, 5'RACE products created by four gene-specific reverse primers (colors indicate the arrows shown on exon sites under the gel figure).

A

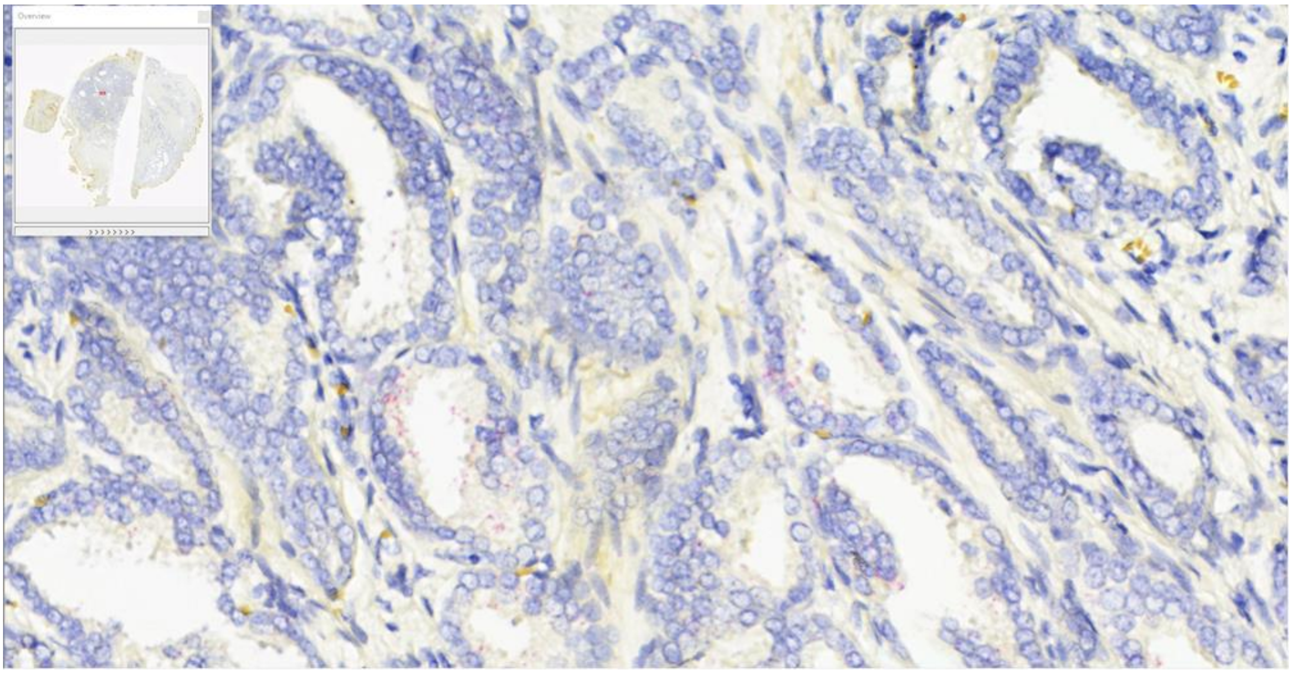

B

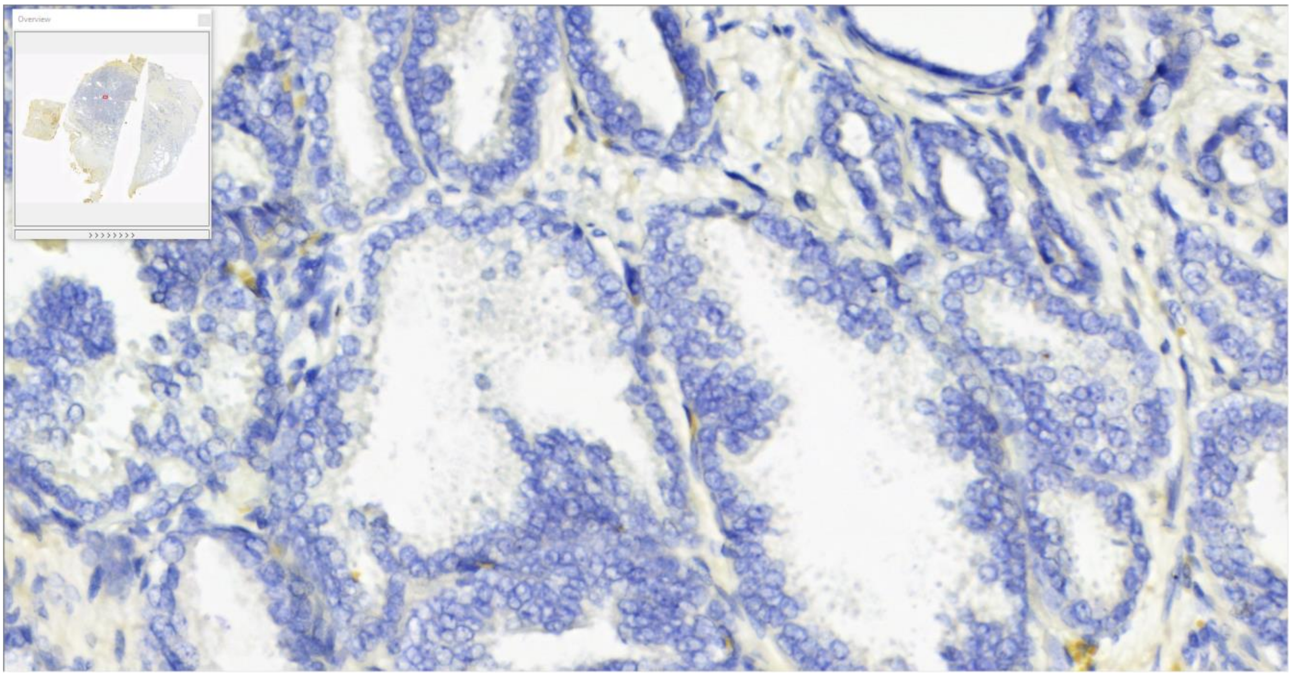

C

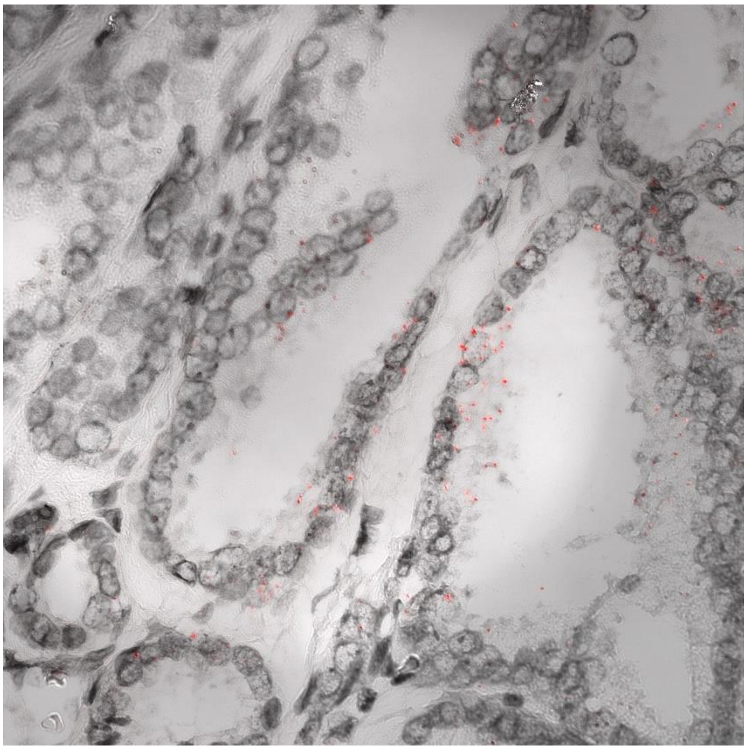

Supplementary Figure 2. *EPCART in situ* hybridization in PC tissue. *EPCART* specificity in RNA *in situ* hybridization experiment (A) was validated by including a negative control (*dapB*) to the experiments (B). The used tissue sections were cut adjacently, and the images in A and B are taken from the same tissue area. C) High resolution image of *EPCART in situ* hybridization in the PC sample. The sample slide (same as in Figure 1C) was imaged with a brightfield slide scanner (A-B) and a confocal microscope (C). Signal from *EPCART* transcripts were detected with Fast Red and the slides were counterstained with hematoxylin.

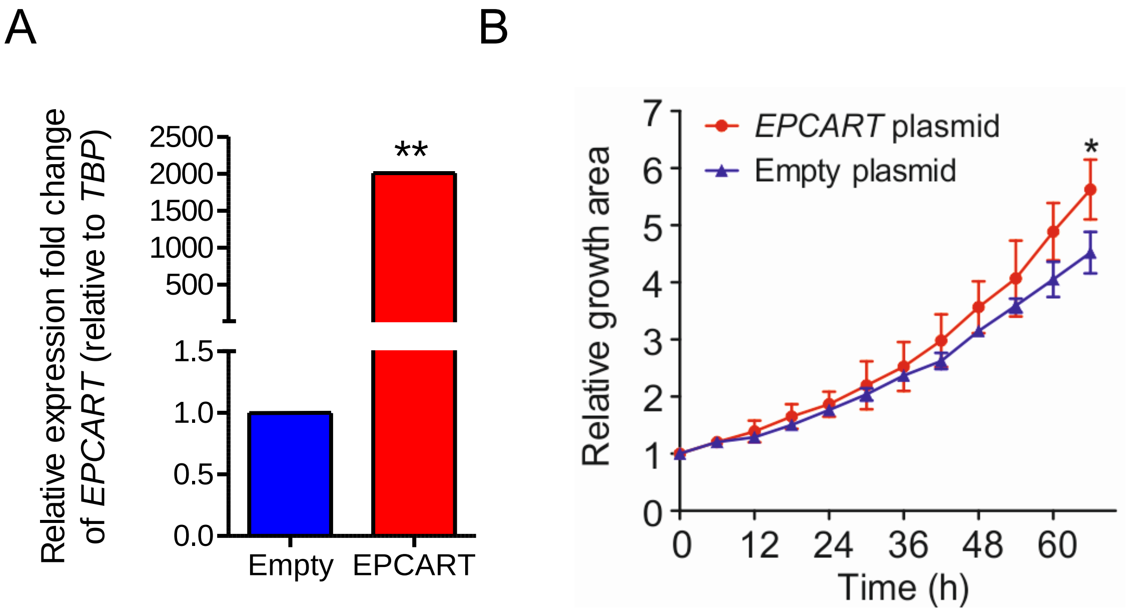

Supplementary Figure 3. Effect of *EPCART* overexpression on prostate cancer cells. A) Expression analysis of *EPCART* in LNCaP cells stably overexpressing *EPCART* or the empty plasmid. Expression was analyzed by qRT-PCR in two biological replicates using *TBP* as a reference gene. Relative expression values ($2^{-\Delta\Delta Cq}$) for two biological replicates were plotted. Expression differences were assessed with an unpaired two-tailed t-test. B) The proliferation of LNCaP cells stably overexpressing *EPCART* or the empty plasmid as a negative control was measured with a Cell-IQ time-lapse imaging system. Error bars, SD; *, $p < 0.05$; **, $p < 0.01$; ***, $p < 0.001$; data were assessed with an unpaired two-tailed t test.

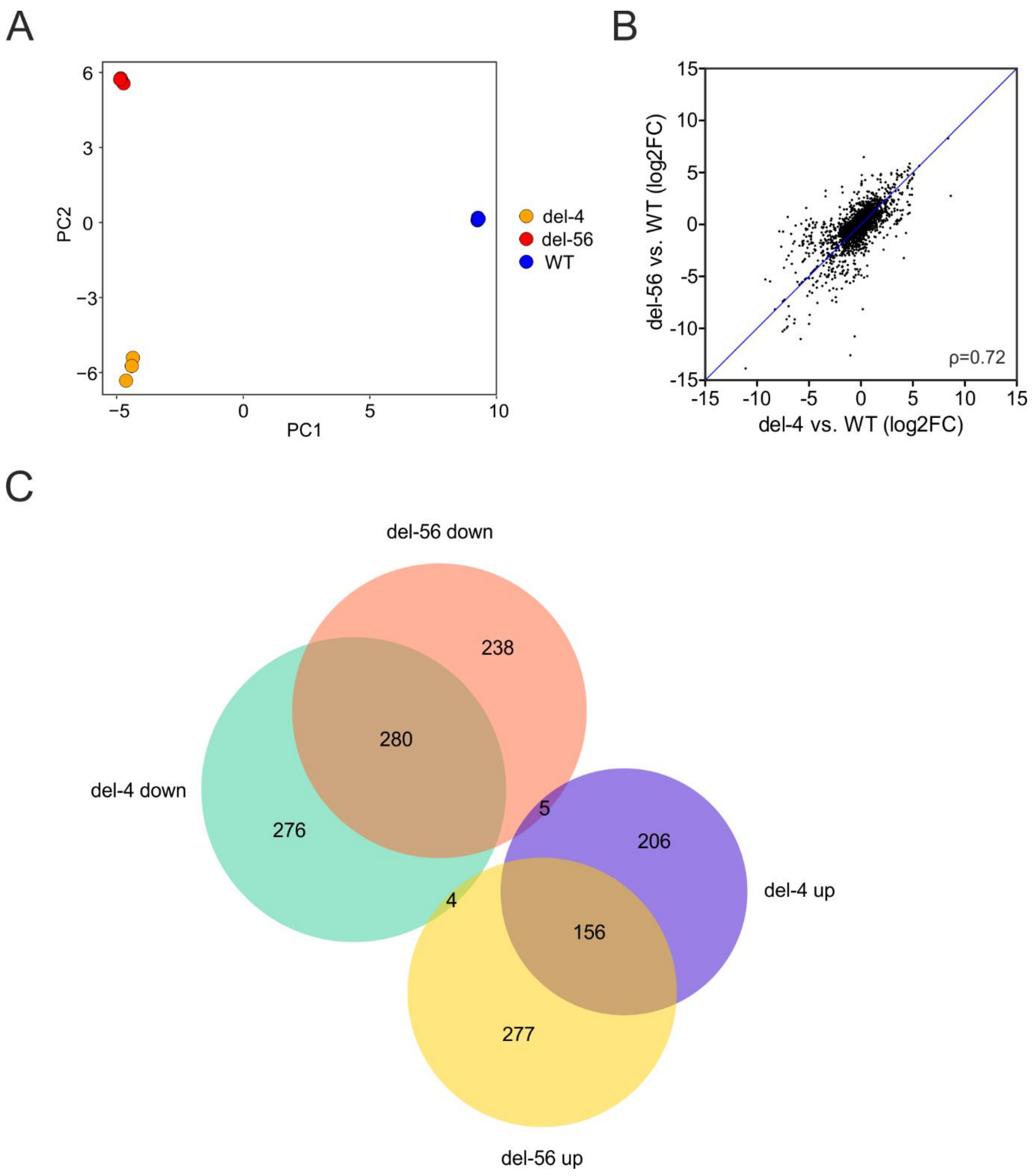

Supplementary Figure 4. Analysis of RNA-seq data of *EPCART*-del cells. A) Principal component analysis of expression three biological replicates of each *EPCART*-del (del-4 and del-56) and WT clone. B) Comparison of all significant $\log_2$FC values (p<0.05) of protein-coding genes between *EPCART*-del clones. Strong positive correlation (Pearson correlation coefficient, ρ) was detected between the clones. The blue line represents perfect positive correlation (ρ=1). C) Venn diagram of differentially expressed genes in *EPCART*-del cloned. Shared upregulated ($\log_2$FC >2) and downregulated ($\log_2$FC <-2) genes between del-4 vs. WT and del-56 vs. WT are shown.

A

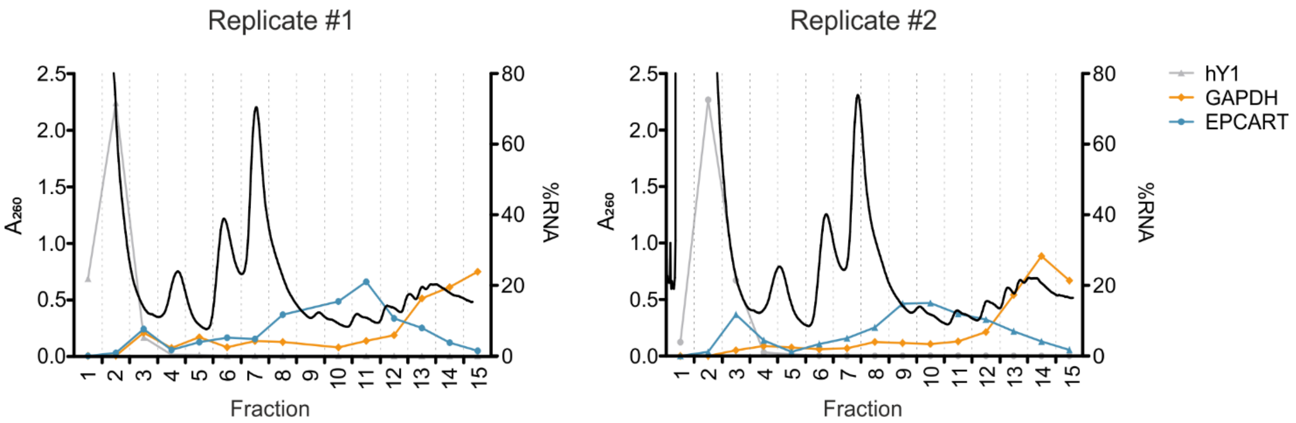
Replicate #1
Replicate #2
A260
%RNA
Fraction
hY1
GAPDH
EPCART

B

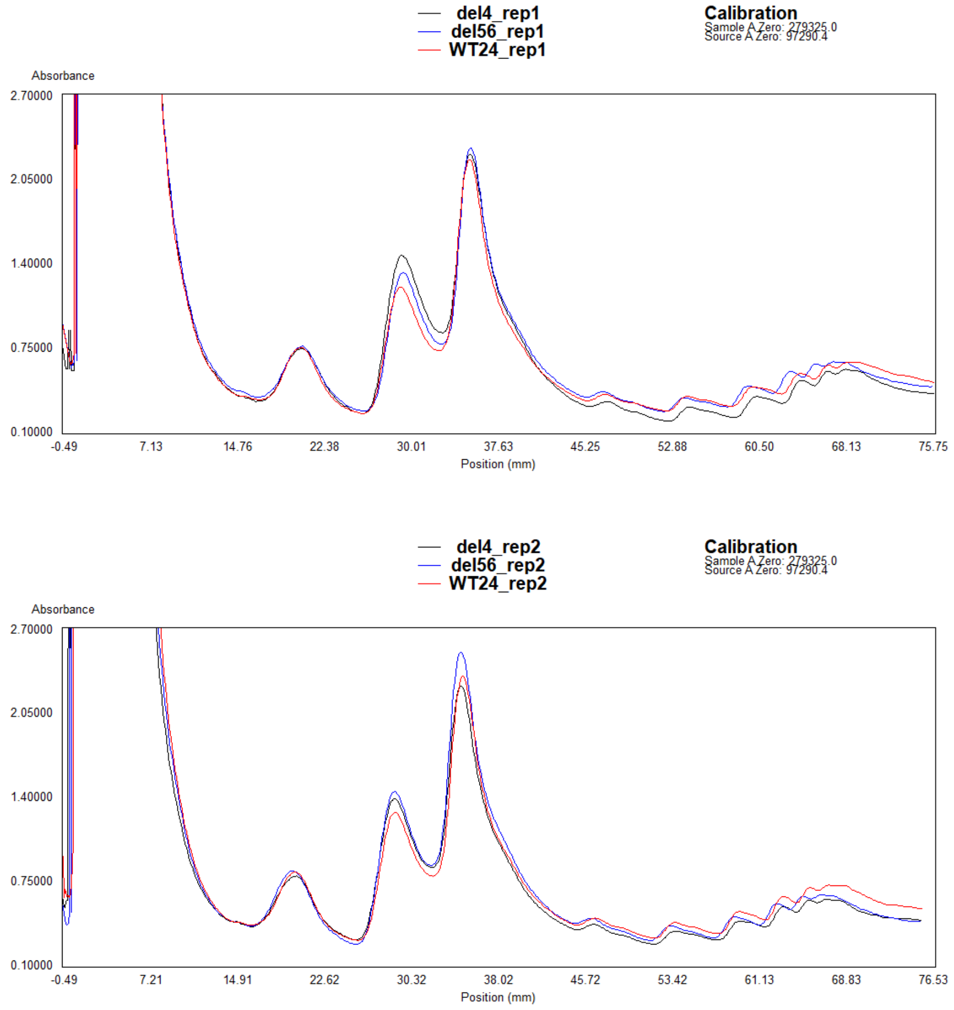
del4_rep1
del56_rep1
WT24_rep1
Calibration
Sample A Zero: 279325.0
Source A Zero: 97290.4
Absorbance
Position (mm)
del4_rep2
del56_rep2
WT24_rep2
Calibration
Sample A Zero: 279325.0
Source A Zero: 97290.4
Absorbance
Position (mm)

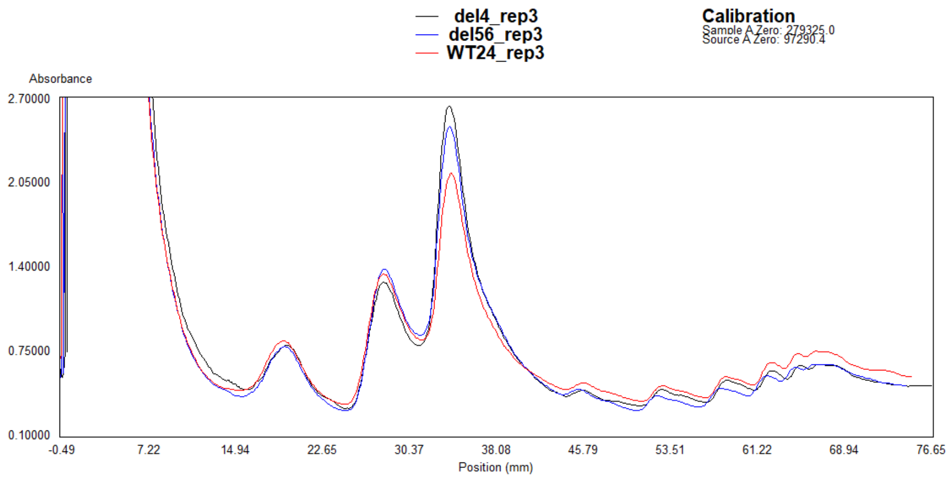

Supplementary Figure 5. Polysome profiling results. **A**) RNA percentage of *EPCART* in polysome profiling fractions. Polysome profiling was done to WT clones (LNCaP). RNA was extracted from each fraction and analyzed by qRT-PCR. %RNA of each fraction was calculated for EPCART, GAPDH (positive control), and hY1 (negative control), and plotted in the same graph with the polysome profile (A_{260}). Graphs presents two different replicates. **B**) Polysome profiles of *EPCART*-del and WT clones for three biological replicates. Replicates with the same number were processed at the same time. The polysome profiles of each replicate group were overlapped.

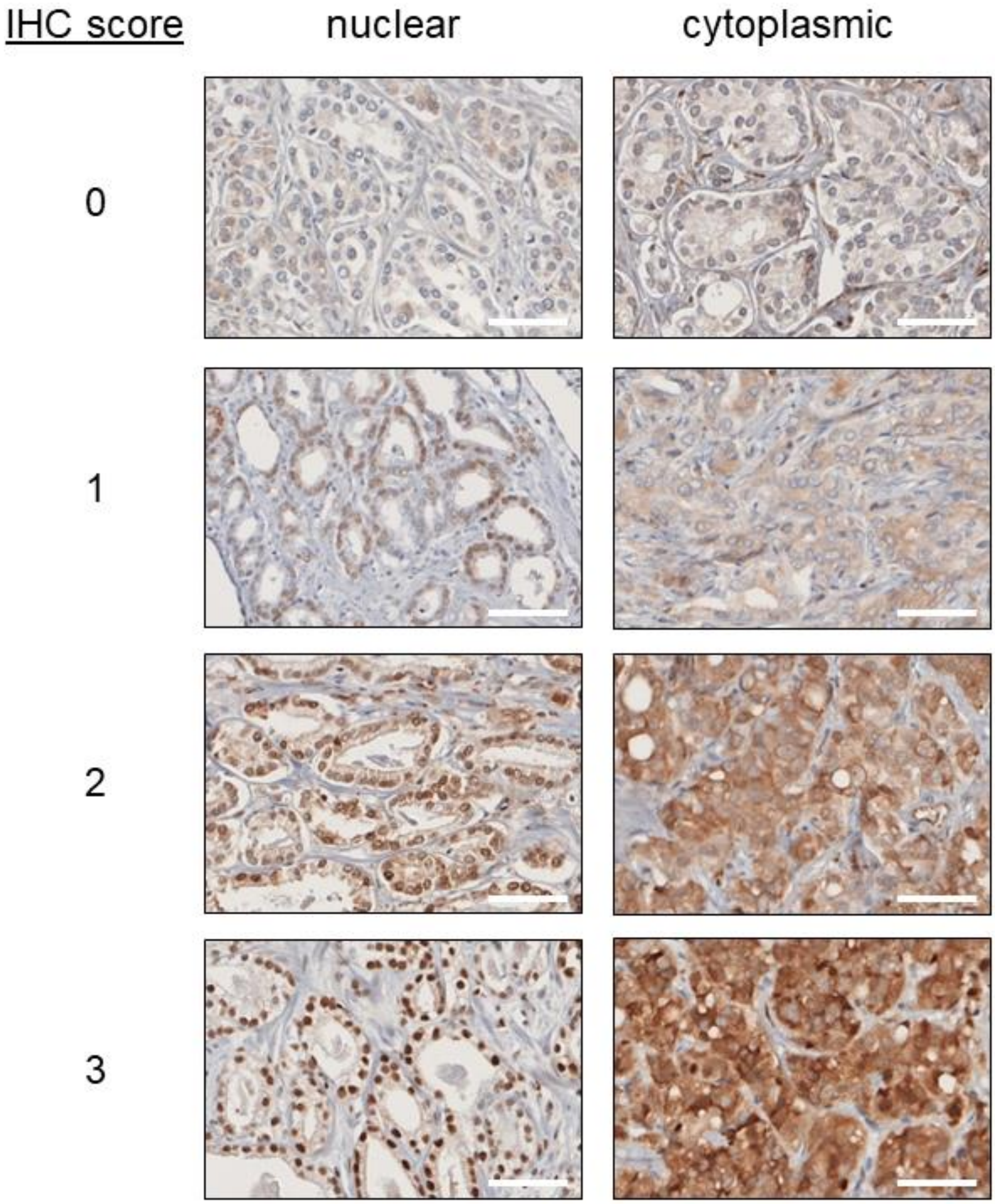

Supplementary Figure 6. Representative images from PCa tumor samples displaying scores 0 to 3 for nuclear and cytoplasmic immunohistochemical staining of PDCD4. White scale bar, 100 µm.
